# The Frequency of Vitamin D and Calcium Deficiencies Among Women of Reproductive Age in Wadi Etba, Southern Region of Libya

**DOI:** 10.7759/cureus.29832

**Published:** 2022-10-02

**Authors:** Annour M Alalem, MYG Younis, Salima M Hawda, Ahmed M Zakoko, Negia A Mohamed, Khalid G Alqathafy, Mohammed O Elmansouri

**Affiliations:** 1 Department of Public Health, Fezzan University, Wadi Etba, LBY; 2 Department of Biochemistry, University of Benghazi, Benghazi, LBY

**Keywords:** vd photosynthesis, wadi etba, hypovitaminosis, total calcium, vitamin d

## Abstract

Background

Vitamin D (VD) is the sunshine vitamin. Its deficiency is widely spread worldwide and is implicated in various health problems that have serious financial, mental, and physical health burdens. In Libya, it also has a high incidence as many studies have been conducted on this issue, but the exact situation of VD deficiency in the southern region of Libya remains unclear.

Aim

The aim of this study is to investigate the frequency of VD deficiency and calcium status among females of reproductive age in Wadi Etba (located in the southern region of Libya).

Material and methods

This study included 622 females aged 20-40 years attending the Rural Hospital and Private Clinics in Wadi Etba for various health issues during the last five years. The study population was further subdivided into two age groups; 20-30 years and 31-40 years. VD and calcium were measured to determine the VD status among the study population.

Results

In general, 489 females had sub-optimal VD (<30 ng/mL), representing 78.6% of the total subjects. Normal VD levels were represented only by 133 subjects (21.4%). VD deficiency was represented by 354 subjects (57%) of the total subjects. A total of 232 (37%) subjects were VD-deficient females (18.3 ± 5.5 ng/ml), 122 subjects (20%) were considered poor (severe deficient, VD <10 ng/ml) represented with a mean serum VD of 8.2 ± 0.6. Furthermore, deficiency cases dominated the older age group. Regarding calcium (Ca^+2^) levels, 31% had lower than the normal range, 68% had normal range, and only 1% showed high Ca^+2 ^levels. On the other hand, there was no correlation between serum levels of VD and Ca^+2 ^levels.

Conclusion

VD deficiency has become a widespread condition in the southern region of Libya. Therefore, we recommended making lifestyle changes, including extending the exposure time to the sunlight radiation, fortifying foods and drinks with VD, and taking supplementary doses of VD to reduce the high incidence of VD deficiency.

## Introduction

Vitamin D (VD) (1,25 hydroxycholecalciferol) is essential for maintaining calcium hemostasis and bone structure. It is well known that VD deficiency causes rickets in children and osteomalacia in adults [1]. However, normal VD levels exert many non-skeletal influences that play a vital role in many health-related conditions. These include pregnancy and birth outcomes, heart diseases, different types of tumors, diabetes, respiratory diseases, and many other conditions related to increased mortality rates [2]. In support of these facts, a recent work found that elevating the levels of VD twice the baseline level in several chronic and inflammatory diseases, such as type 2 diabetes mellitus (T2DM), Alzheimer's disease, cardiovascular disease (CVD), and cancer, led to a reduction in mortality rate by 11% in the East of Mediterranean area. A two years increase in life expectancy was also noted [3].

VD is also called the sunshine vitamin because 80% of VD formed under the skin is by the direct effect of sunshine (mainly ultraviolet B radiation with a wavelength of 290-320 nanometers). It penetrates the skin and alters 7-dehydrocholesterol to pre-vitamin D3, transforming it into active VD3 [4]. In accordance, sun radiations provide a considerable degree of protection against deficiency of VD [5]. On the other hand, food provides a small amount of the daily requirement of VD. It is the active form of VD (1,25-dihydroxyvitamin D3 or 1,25(OH)2D3) which performs the vast majority of the actions of VD. Like steroid hormones, VD is lipophilic; this character enables it to pass through the cell membrane and bind to its nuclear receptor on DNA to alter gene expression of a wide variety of genes through which it exerts its net effects. A good example was the work of Hossein-Nezhad A et al. [6], who showed the effects of altering gene expression in leukocytes following the intake of supplementary VD in obese patients who have cancer.

The serum level of 25-hydroxyvitamin D3 (25(OH) D3; circulating form) is utilized in the measurements of the serum levels of VD. 25(OH)D is further hydroxylated to the active form (1,25(OH)2D3) in kidney cells. In clinical practice, it is difficult to define the deficiency status during pregnancy stages, as suggested by Nassar N et al. [7]. However, there is a high prevalence of VD insufficiency among pregnant women in different countries. The highest one (80%) was reported in the Netherlands, in which VD in pregnant women was less than 25nmol/l (<10ng/ml; severe deficiency), followed by 25% in UAE and about 18% in the UK [8].

VD deficiency is linked to an elevated risk of fatigue and unreasonable pain in the skeleton. In turn, these symptoms considerably affect work duties and social life [9]. In addition, in a recent study, the reduced levels of VD during the pregnancy period have been related to a higher risk of Cesarean delivery, preterm birth, and preeclampsia [10, 11].

Furthermore, VD deficiency is also linked to an increase in the risk of developing gestational diabetes, which may develop into type 2 diabetes, as concluded by the study by Triunfo S et al. in 2017 [12]. The decrease in VD concentrations in women in Libya may be due to outdoor traditional and Islamic dressing, as covering the head with Hijab and or Niqab is very common among Libyan females. In the current work, we investigated serum VD and calcium to find out about the normal, deficiency, and insufficiency situations of VD among Libyan females in Wadi Etba.

## Materials and methods

The study was conducted in the Wadi Etba area, which consists of nine villages situated in the southwest of Libya, about (180 Km south of Sebha, the third largest city in Libya). The total population was about 22,000 inhabitants.

Study population

A total of 622 samples were collected from Libyan women aged between 20 and 40 years attending the medicine department of Tasawah Rural Hospital and Private Clinics during the last five years (between January 2017 to December 2021). The study population was further subdivided into two age groups; 20-0 years (255 subjects: 36.2%) and 31-40 years (397 subjects: 63.8%) (Table 1).

**Table 1:** Distribution of the study subjects into two age groups.

Age groups	Frequency	Percent %
20-30 years	225	36.2
31-40 years	397	63.8
Total	622	100.0

Sample collection and investigations

A total of 5 ml of venous blood was collected from each woman in plain tubes in Al-Abedin medical laboratory. The blood samples were centrifuged at 4000 rpm for 5 minutes, and the serum samples were tested for VD and total calcium. VD was assessed by using a quantitative test of a total of 25(OH)D2/D3 levels in human serum (Ichroma Korean company). Based on the Endocrine Society Clinical Practice Guideline on the treatment and prevention of VD, the recommended deficiency and insufficiency levels of VD [25(OH)D] are illustrated, as shown in Table 2. 

**Table 2:** The normal, deficiency, and insufficiency range of vitamin D in human serum. This table was adapted from https://www.robertbarrington.net/.

Blood levels (ng/ml)	VD status
Less than 10 ng/ml	Poor (Severe deficiency)
10-20 ng/ml	Deficiency
20-30 ng/ml	Insufficiency
More than 30 ng/ml	Normal
Over 100 ng/ml	Overdose

Total calcium in serum was evaluated by using a photometric test applying the o-cresolphthalein complexone (CPC) method from HUMAN (a German company).

Statistical analysis

The results were analyzed using SPSS version 20.0. The Chi-square test was used to measure the statistically significant differences at p<0.05.

## Results

The mean ± SD of the age of the study subjects was 32.5 ± 5.4 years. The range (lowest-highest values) of serum levels of VD and calcium (Ca^+2^) were 5-80 ng/ml and 4.2-11.3 mg/dl, respectively. The mean ± SD of serum VD and Ca^+2^ of the study subjects was 21.9 ± 14.3 ng/ml and 8.5 ± 0.9 mg/dl, respectively (Table 3).

**Table 3:** Mean and SD of age, vitamin D, and calcium of the study population.

Parameter	N	Minimum	Maximum	Mean	SD
Age	622	20.00	40.00	32.5	5.45
Vitamin D	622	5.00	80.00	22	14.3
Calcium	622	4.20	11.30	8.5	0.9

Distribution of VD according to concentration

generally, 489 females have sub-optimal VD, representing 78.6% of the total subjects. Normal VD levels are represented only by 133 subjects (21.4%). Based on serum concentration of VD, it is distributed into normal, insufficient, deficient, and poor (severe deficiency), as shown earlier in the Material and methods section (Table 1). A total of 133 subjects out of 622 (21.4%) showed normal (>30 ng/ml) serum VD levels (mean ± SD; 44.83 ± 11.9), as seen in Table 4. A total of 232 subjects (37%), representing the largest group of the study population, showed deficiency in serum levels of VD (10-20 ng/ml), with a mean of 18.3 ± 5.5 ng/ml. Poor (severe deficiency; VD is <10 ng/ml) represents 122 subjects (20%) with a mean serum VD of 8.2 ± 0.6. The VD insufficiency (20-30 ng/ml) represents 135 subjects of the study population (22%) with serum VD levels of 24.51 ± 2.77 ng/ml.

**Table 4:** VD status among the study population. VD: Vitamin D.

VD status	N	Percentage	Mean ± SD	Range (ng/ml)
Poor	122	20%	8.2 ± 0.6	5-9.9
Deficiency	232	37%	14.7 ± 2.8	10-19.8
Insufficiency	135	22%	24.5 ± 2.8	20-30
Normal	133	21.4%	44.8 ± 11.9	30-80

Distribution of serum calcium according to concentration

According to Ca^+2^ serum concentration, 425 subjects showed normal serum levels; 8.86 ± 0.61 mg/dl (68.3%; the highest percentage of study population had normal Ca^+2^ levels), 189 subject (34.2%) showed lower Ca^+2^ levels (7.47 ± 0.19 mg/dl) and only eight subjects (1.3%) showed Ca^+2^ levels higher than the normal levels 10.9 ± 0.19 mg/dl, as seen in (Table 5).

**Table 5:** Ca+2 status in the study subjects.

Serum Ca^+2 ^Levels	N	Mean ± SD	Percentage%
Lower than normal	189	7.47 ± 0.48	31%
Normal	425	8.86 ± 0.61	68%
Higher than normal	8	10.9 ± 0.19	1%

Correlation of VD and calcium levels with age groups

The mean ± SD of VD in the age group 31-40 years was significantly increased (p < 0.045*) compared to VD and the age group 31-40 years. But there was no significant difference between calcium levels in both age groups (Table 6). Moreover, the correlation coefficient between the serum levels of VD and the serum Ca^+2^ levels was non-significant (Table 7).

**Table 6:** Correlation between VD and calcium with the age groups. VD: Vitamin D.

Parameters	Age group	N	Mean ± SD	Sig.	Decision
VD	20-30	225	20.5 ± 12.5	0.045*	Significant
	31-40	397	22.8 ± 15.2		
Ca^+2^	20-30	225	8.4 ± 0.9	0.189	Not Significant
	31-40	397	8.5 ± 0.9		

**Table 7:** Correlations between VD and Ca+2. VD: Vitamin D.

Parameters		VD	Ca^+2^
VD	Pearson Correlation	1	-0.001-
	Sig. (2-tailed)		0.984
	N	622	622
Ca^+2^	Pearson correlation	1	-0.001-
	Sig. (2-tailed)		0.984
	N	622	622

Crosstabulation of VD groups and the age groups

Most normal VD cases were shown in the 31-40 years age group (93 subjects, 69.9%), whereas the 20-30 years age group showed only 40 subjects (30.1%), as shown in Table 8. The vast majority of VD deficient subjects (143 subjects) were found in the 31- 40 years age group, while 89 VD deficient subjects (38%) were found in the 20-30 years age group. Most of the poor (severely deficient) serum VD cases were found among the 31-40 years age group with 73 subjects (59.8%), whereas the 20-30 years group had only 49 subjects (40.2%) who presented with poor VD. In the case of VD insufficiency, it appeared in 231 subjects (62.9%) within the age group 31-40 years, while the age group 20-30 years showed 136 subjects (37.1%) who presented with VD insufficiency.

**Table 8:** Crosstabulation of classes of Vitamin D status and age groups.

Classes of Vitamin D		Age (years)		Total
		20-30	31-40	
Poor	Count	49	73	122
	%	40.2%	59.8%	100.0%
Deficiency	Count	89	143	232
	%	38%	62%	100%
Insufficient	Count	136	231	367
	%	37.1%	62.9%	100.0%
Normal	Count	40	93	133
	%	30.1%	69.9%	100.0%

Crosstabulation of VD groups and calcium levels

As clear from Table 9, most subjects with various VD statuses had normal Ca^+2^. In the poor (severe deficiency) VD group, there were 37 subjects who had serum Ca^+2^ levels lower than normal, 84 subjects had normal Ca^+2^ levels, and only 21 cases had high Ca^+2^ levels. In the deficiency group, 72 subjects presented with sub-normal Ca^+2^. In most of the cases, 158 subjects (out of 232 subjects) had normal Ca^+2^, and only two subjects had high Ca^+2^. In the insufficient VD group, 38 subjects had Ca^+2^ levels lower than normal, 95 subjects had normal Ca^+2^ levels, and only two cases had high serum Ca^+2^. In the normal VD group, 42 subjects had Ca^+2^ levels lower than normal, 88 subjects had normal Ca^+2^ levels, and only three cases had high Ca^+2^ levels.

**Table 9:** Crosstabulation of Ca+2 and VD classes of the study subjects. VD: Vitamin D.

Parameters			VD status			
			Poor	Deficiency	insufficiency	Normal
Ca^+2^ Status	Ca^+<^< Normal	Count	37	72	38	42
		% within VD	30%	31%	28.1%	31.6%
	Normal Ca^+2^	Count	84	158	95	88
		% within VD	68.8%	68.1%	70.4%	66.2%
	Ca^+2 ^> Normal	Count	1	2	2	3
		% within VD	0.82%	0.86%	1.48%	2.2%
Total		Count	122	232	135	133

## Discussion

In recent years, VD deficiency has become a global problem, almost affecting the whole six continents of the universe despite the availability of sunshine which aid in the photosynthesis of cutaneous VD and/or consumption of supplementary VD doses. In adults in Vietnam, Hien VT et al. [13] reported a prevalence of VD deficiency of 7% in 2009, which is further highly elevated in females to reach the edge of 30% in 2012, as reported by Nguyen HT et al. [14]. In 2013, the prevalence became almost twice (57%) reported in the previous year [15]. The latter finding was equal to the figure of VD deficiency (57%; for both poor and deficient VD) reported by the present study. 

The golden standard test for diagnosing VD deficiency is 25-hydroxyvitamin D. The vast majority of scientists and researchers in this field decided that serum levels of VD below 20 ng/ml indicate VD deficiency. At the same time, VD levels lower than 10 ng/ml are considered severely deficient (poor). When the VD levels are lower than 30 ng/ml (20-30 ng/ml), it is called VD insufficiency [16].

In the present work, out of 622 women who were recruited, 232 subjects (37%) were categorized as having VD deficiency. At the same time, the severely deficient VD percentage was 20% (122 subjects with a VD level of 8.2±0.6 ng/ml). Therefore, the normal VD in serum (44.8 ± 11.88 ng/ml) was represented by only 133 women (21.4%), which is two times less than the prevalence of deficient VD women (57%) in the current work. This finding agrees with previous work in Libya, which showed a higher prevalence (76%) of VD deficiency in Libyan women in Benghazi, the second largest city in Libya, by Omar M et al. [17]. Furthermore, Nasef A et al. [18] reported that about 63% of the cases attending different clinics in the central region of Libya were suffering from VD deficiency.

On the other hand, Al‑Graiw MH et al. [19] investigated 262 female subjects suffering from nonspecific musculoskeletal and bone pain at Seoul Hospital in Tripoli, Libya. They found that 50.8% showed severe VD deficiency (VD serum levels<10 ng/ml) which is more than double the rate of severe deficiency of VD in the current study (20%). Furthermore, the present work showed that 37% of the study population had VD deficient levels, which was higher than the results of the last evidence, which found only 27.6% presented with VD deficiency (VD levels <20 ng/ml). These studies were in accordance with previous work in Libya, which demonstrated that serum VD levels of less than 10 ng/mL is used as a marker of severe VD deficiency [20, 21].

There is a big gap in VD research studies between the northern coastal cities in Libya and the rural areas and villages. This study was conducted to minimize this knowledge gap about VD deficiency in rural areas in Libya, such as Wadi Etba. Women are more susceptible to VD deficiency than men in rural areas. The first observational remark is that the prevalence of rate of VD deficient women was about twice the normal VD; 37% and 21%, respectively, while the prevalence rate of severely deficient (poor) VD is about the same as the rate of normal VD 20% and 21%, respectively. These findings were in the same line with a recent study in the coastal city of Benghazi (the second largest city in Libya), in which serum VD levels were tested in 184 subjects recruited from three polyclinics in Benghazi. Most of them were females (58.8% females and 5.9% males). In this study, Omar M et al. reported that VD deficiency was diagnosed in about 76.1% (3/4 of the studied subjects), whereas 15.2% presented with insufficient serum VD, and only 8.7% showed normal VD levels [17].

In the current work, the mean serum levels of VD were significantly (p < 0.045*) increased in all the females of the age group (31-40 years) compared to the mean serum VD in the females within the age group of 20-30 years. On the other hand, the current study assessed the distribution of different categories of VD status and found that the majority of the poor VD was found within the older age group (73 subjects, 59%). In the same line, the VD deficient and VD insufficient females were found within the 31-40 years age group (143 subjects, 62%, 230 subjects, 62.9%, respectively), which indicates that VD deficiency is more prevalent in the older adult females. These findings were in the same line with the work of Nasef A et al. [18]. They found that the incidence of VD deficiency existed maximally in the older age and also reported that being a female is regarded as a risk factor for developing sub-optimal levels of VD. Our suggestion is in agreement with Omar M et al. [17], who found that VD deficiency gets worse with an increase in age (e.g., in the age 20 and 40 years, VD level was 13.9 ng/ml compared with higher levels reported in younger ages).

As shown in the current study, the Ca^+2^ levels that are lower (7.47 ± 0.48 mg/dl) than the normal cutoff value represent 31% of the subjects, however crosstabulation of Ca^+2^ against VD revealed that most of the poor, deficient, and insufficient VD cases had normal Ca^+2^ levels (68.8 %, 68.1 and 70.4%, respectively). In contrast, the lower Ca^+2^ levels represent only 30%, 31%, and 28.1%. Furthermore, the correlation of serum VD levels with Ca^+2^ levels was insignificant. In contrast, Al-Graiw MH et al. [19] demonstrated a significant positive correlation between Ca^+2^ and VD. In accordance with Al-Graiw MH et al., Nair R and Maseeh A [22] suggested that VD deficiency is associated with reduced Ca^+2^ levels. Recent evidence reported a reduction in intestinal absorption of Ca^+2^ in older age, which may lead to secondary hyperparathyroidism and bone demineralization [23]. On the contrary, our study showed that Ca^+2^ levels were not affected by increasing age, and no significant change was found by comparing the Ca^+2 ^levels in both of the age groups; 20-30 years and 31-40 years.

Exposure to sunlight is one of the primary sources of supplying VD for humans [24]. Therefore, it is recommended to expose oneself to sunlight between 10 AM and 3 PM daily for 5-15 minutes. The arms, legs, hands, and face should be uncovered to receive solar radiation. The decrease in VD concentrations in women in Libya and Middle Eastern countries despite the sun shining all year may be due to outdoor traditional and Islamic dressing. There are two styles of dressing in Libya, the first is called Hijab, which covers the entire body except for the face and hands, and the other is called Niqab, which covers all the body, including the face. These styles provide a reasonable explanation for the association between VD deficiency and the female gender in Libya and other Arabic countries. These dressing styles caused a reduction in the synthesis of VD by photosynthesis by preventing the sunshine radiation from reaching the skin. This suggestion was supported by the work of Al‑Graiw MH et al., who reported reduced serum levels of VD in Libya compared with the serum VD levels in females wearing westernized style clothes. This finding is supported by the work of Perampalam S et al., who reported that VD production increased with the increasing exposure of larger areas of skin to sunshine radiations [25]. This finding indicated that covering the body with extra clothing can markedly diminish the photosynthetic synthesis of VD [26].

Among the factors that contribute to VD deficiency; is the dietary behavior in Libya. Most of the food and drinks in the market are not VD-fortified food. In order to overcome this issue, a VD supplement of 1000 IU should be given to individuals at risk, especially in the winter. Furthermore, recent work reported that omega-3 and cod liver oil supplement has a protective ability against VD deficiency (Nasef A et al.). This finding is in line with the works of Fields J et al. and Lee SM et al., who suggested that VD deficiency is also linked to obesity and overweight [26-27]. The excess fat in the body may reduce VD absorption.

## Conclusions

As clear from this study, sub-optimal and deficiency of VD have become a widespread condition in the Southern region of Libya. VD deficiency also has a high prevalence in other regions of Libya and other areas of the world, making it a challenging issue for health professionals. The causes of the widespread VD deficiency are not entirely understood. As it is clear from VD research, VD deficiency is linked to many health problems, including autoimmune disease, cardiovascular diseases, diabetes, and cancer. This suggests that taking preventive measures becomes a necessity. We recommend making lifestyle changes such as extending the exposure time to sunlight radiations. We are not recommending that Muslim ladies remove their Hijab, but we advise them to try to find ways to expose their hands, arms, legs, and faces to the sunlight to get VD. Other life changes include fortifying foods and drinks with VD and taking vitamin supplements as well as omega-3 and cod liver oil, which contain a considerable amount of VD, which may play a vital role in avoiding VD deficiency.
